# Selection and Regulatory Network Analysis of Differential CircRNAs in the Hypothalamus of Goats with High and Low Reproductive Capacity

**DOI:** 10.3390/ijms251910479

**Published:** 2024-09-28

**Authors:** Shuaixiang Mao, Cuiying Wu, Guanghang Feng, Yaokun Li, Baoli Sun, Yongqing Guo, Ming Deng, Dewu Liu, Guangbin Liu

**Affiliations:** College of Animal Science, South China Agricultural University, Guangzhou 510642, China

**Keywords:** Chuanzhong black goat, hypothalamus, prolificacy, circRNA

## Abstract

The objectives of this investigation were to identify differentially expressed circular RNAs (circRNAs) in the hypothalamus of goats with high and low prolificacy and construct a circRNA-mRNA regulatory network to uncover key potential circRNAs that influence goat prolificacy. Transcriptome analysis was performed on hypothalamus samples from low-prolificacy (*n* = 5) and high-prolificacy (*n* = 6) Chuanzhong black goats to identify circRNAs that influence prolificacy in these goats. Differential expression analysis identified a total of 205 differentially expressed circRNAs, comprising 100 upregulated and 105 downregulated circRNAs in the high-prolificacy group compared with the low-prolificacy group. Enrichment analysis of these differentially expressed circRNAs indicated significant enrichment in Gene Ontology terms associated with mammalian oogenesis, negative regulation of neurotransmitter secretion, reproductive developmental processes, hormone-mediated signaling pathways, and negative regulation of hormone secretion. Kyoto Encyclopedia of Genes and Genomes (KEGG) analysis highlighted significant enrichment in the oxytocin signaling pathway, GnRH signaling pathway, and hormone-mediated oocyte maturation. The hypothalamus of low- and high-prolificacy goats contains circular RNAs (circRNAs), including chicirc_063269, chicirc_097731, chicirc_017440, chicirc_049641, chicirc_008429, chicirc_145057, chicirc_030156, chicirc_109497, chicirc_030156, chicirc_176754, and chicirc_193363. Chuanzhong black goats have the potential to influence prolificacy by modulating the release of serum hormones from the hypothalamus. A circRNA-miRNA regulatory network was constructed, which determined that miR-135a, miR-188-3p, miR-101-3p, and miR-128-3p may interact with differentially expressed circRNAs, thereby regulating reproductive capacity through the hypothalamic-pituitary-gonadal axis. The results of this study enhance our knowledge of the molecular mechanisms that regulate prolificacy in Chuanzhong black goats at the hypothalamic level.

## 1. Introduction

Goats possess unique advantages in environmental adaptability and functional diversity. Goat farming is considered a relatively low-investment venture compared with farming other livestock due to their body size, productivity levels, dietary preferences, and production costs, all of which are favorable for human use [1]. Goats do not compete with humans for food resources and can provide abundant meat, milk, fur, and other products, which is why they have become one of the most widely distributed livestock species globally [2]. The Chuanzhong black goat, a local breed found in central shallow hilly areas and adapted to high-altitude climates, exhibits rapid growth, excellent meat quality, strong adaptability, and resilience to coarse forage [3]. Kid production is a crucial reproductive trait in goats, directly impacting their economic viability. Therefore, breeding goat breeds with high reproductive rates is essential for developing the goat industry [4,5]. The Chuanzhong black goat population includes groups with both high and low reproductive rates. Utilizing high-throughput sequencing techniques to identify genes associated with kid production traits in Chuanzhong goats can provide valuable insights into the genetic factors influencing goat prolificacy.

The ovary is a reproductive organ in female animals. In the estrous cycle of goats, the number of mature oocytes released by the ovary is a crucial factor influencing goat kid production. Ovarian function is regulated by the hypothalamus and the pituitary gland [6]. The hypothalamus primarily regulates the ovary primarily by secreting gonadotropin-releasing hormone (GnRH), which controls the secretion and release of luteinizing hormone (LH) and follicle-stimulating hormone (FSH) from the anterior pituitary gland. These hormones can bind to receptors in the ovary to regulate the ovulation process [7]. Studies have shown that goats exhibit a surge in LH prior to ovulation [8], indicating that the hypothalamus plays a central role in the reproductive control process in goats. The synthesis and secretion of GnRH in the hypothalamus may be influenced by non-coding RNA. However, research on the molecular mechanisms through which the hypothalamus affects goat prolificacy is relatively limited.

Circular RNA (circRNA) is a biologically active nucleic acid molecule that, unlike mRNA, lacks a polyadenylated tail [9,10]. Current research has identified six primary functions of circRNA [11]. The first function is its ability to localize the synthesis site and the host gene, thereby upregulating exons or truncating transcripts [12,13]. The second function of EIciRNA is its capacity to bind to U1 small nuclear ribonucleoprotein, interact with PolII, and enhance the expression of the parent gene [14]. The third function is that it acts as a miRNA sponge, competitively binding miRNA [15]. The fourth function is its ability to interact with proteins [16]. The fifth function is that if circular RNA contains an internal ribosome entry site (IRES), then it can recruit ribosomes and undergo translation [17]. The sixth function is that circRNA containing N6-methyladenosine (m6A) can be recognized by YTHDF3, thereby triggering the translation process [18]. Whole transcriptome analysis can identify both coding and non-coding RNA, quantify the heterogeneity of gene expression across tissues and organs [19], and enhance our understanding of the regulatory relationships between genes [20]. Therefore, utilizing transcriptome sequencing technology to investigate genes associated with reproduction in the hypothalamus is crucial for comprehending the molecular mechanisms through which the hypothalamus regulates animal reproduction.

This study employs transcriptome sequencing technology to investigate the hypothalamus of low- and high-prolificacy Chuanzhong black goats, with the aim of identifying differential circRNAs that may influence the reproductive performance of this breed. The objective is to provide insights into the molecular mechanisms regulating reproduction in Chuanzhong black goats and establish a theoretical foundation for the prolificacy traits of this breed.

## 2. Results

### 2.1. Quality Detection of RNA-Seq Sequencing Data

**Figure 1:**
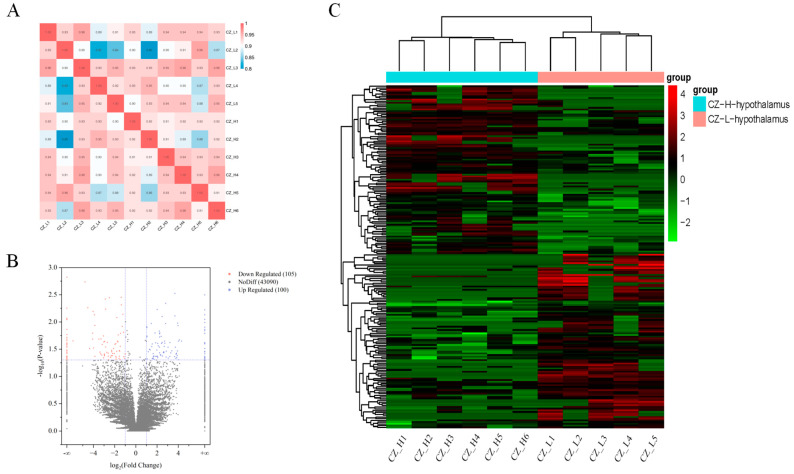
Correlation analysis and differential circRNA profile of hypothalamic samples in Chuanzhong black goats with different reproductive capacities. (**A**) Correlation analysis heatmap of circRNA expression across different samples. (**B**) Volcano plot of differentially expressed circRNAs. (**C**) Heatmap of clustering analysis for differentially expressed circRNAs.

Before further analysis, quality control checks were performed on the raw data obtained from sequencing the hypothalamus samples of the low-prolificacy group (CZ_L) and the high-prolificacy group (CZ_H). Eleven independent cDNA libraries were constructed from the hypothalamic tissue RNA of both the CZ_L and CZ_H groups. A total of 1,142,854,448 raw sequence reads were generated from the 11 sequencing libraries, of which 1,134,036,480 high-quality reads passed quality control and were used for subsequent analysis. The base identification rate for each sample ranged from 92.04% to 93.48%, with more than 99.9% of bases identified correctly. The alignment rates of high-quality reads to the reference genome were above 84.98%. Among these high-quality reads, 1.45% to 2.38% aligned to multiple locations, while 97.62% to 98.55% aligned to unique positions. These data indicate high sequencing quality, fully meeting the requirements for subsequent analysis, as illustrated in Table 1 [21]. The correlation analysis of circRNA expression among the samples is depicted in Figure 1A.

### 2.2. CircRNA Differential Expression Analysis

The hypothalamus, a crucial component of the hypothalamus-pituitary-ovary axis in goats, may influence the reproductive capacity of Chuanzhong goats through the differential expression of genes or transcripts. On the basis of the criteria of |log2 Fold Change| > 1 and a *p*-value < 0.05, we identified differentially expressed circular RNAs (DEcircRNAs) between the low-prolificacy and high-prolificacy groups. With the use of hypothalamic samples from the low-prolificacy group as a control, the volcano plot revealed a total of 43,295 circular RNAs detected in both the low- and high-prolificacy groups. Among these, 205 circRNAs exhibited differential expression, with 100 circRNAs upregulated and 105 circRNAs downregulated (Figure 1B, Supplementary Table S1). Clustering analysis indicated that the gene expression levels and patterns of the six samples from the high-prolificacy group were similar, while the five samples from the low-prolificacy group also displayed comparable patterns (Figure 1C).

### 2.3. Functional Enrichment Analysis of Host Genes with Differentially Expressed circRNAs

**Figure 2:**
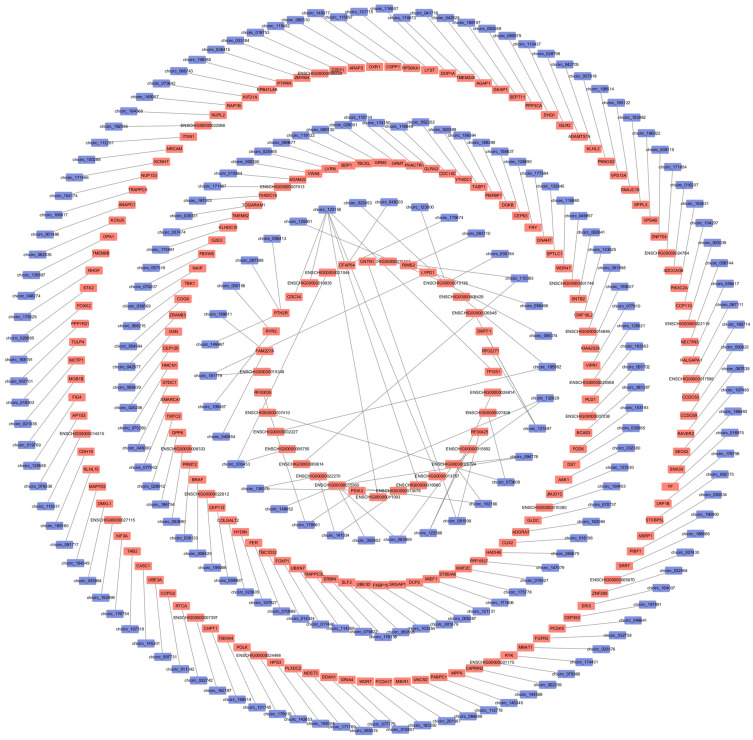
Host gene network map of differentially expressed circRNAs.

**Figure 3:**
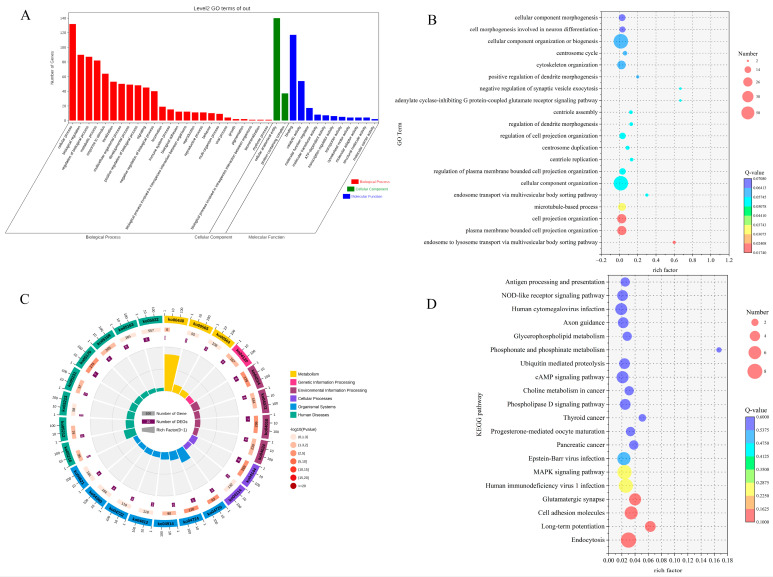
GO and KEGG analysis of differentially expressed circRNA host genes. (**A**) GO annotation involves categorizing genes based on BP, CC, and MFs. (**B**) Bubble plot of GO enrichment for target genes of differentially expressed circRNAs. (**C**) Circular plot of KEGG pathway enrichment for target genes of differentially expressed circRNAs. (**D**) Bubble plot illustrating the KEGG pathway enrichment for target genes of differentially expressed circRNAs.

In our investigation of the functional roles of differentially expressed circRNAs in the hypothalamus of Chuanzhong black goats with varying reproductive capacities, we identified the host genes associated with these circRNAs (Figure 2, Supplementary Table S4) and performed Gene Ontology (GO) functional enrichment analysis on these host genes. Our analysis revealed that the host genes of differentially expressed circRNAs were enriched in 3257 GO terms. A differential significance enrichment analysis of the results identified 486 significantly enriched GO terms (*p* < 0.05), which included 338 biological processes (BP), 59 cellular components (CC), and 89 molecular functions (MF). The four most enriched biological processes were cellular processes, regulation of biological processes, biological regulation, and metabolic processes. The two most enriched cellular components were protein-containing complexes and cellular anatomical entities. The four most enriched molecular functions were binding, catalytic activity, molecular function regulation, and ATP-dependent activity (Figure 3A,B, Supplementary Table S2).

For the host genes of differentially expressed circRNAs, Kyoto Encyclopedia of Genes and Genomes (KEGG) enrichment analysis was performed, identifying enrichment in 183 pathways, of which 15 were significantly enriched (*p* < 0.05). These pathways include progesterone-mediated oocyte maturation, the MAPK signaling pathway, the cAMP signaling pathway, and the phospholipase D signaling pathway (Figure 3C,D, Supplementary Table S3).

### 2.4. Analysis of the Regulatory Network of Differential circRNAs and miRNAs

**Figure 4:**
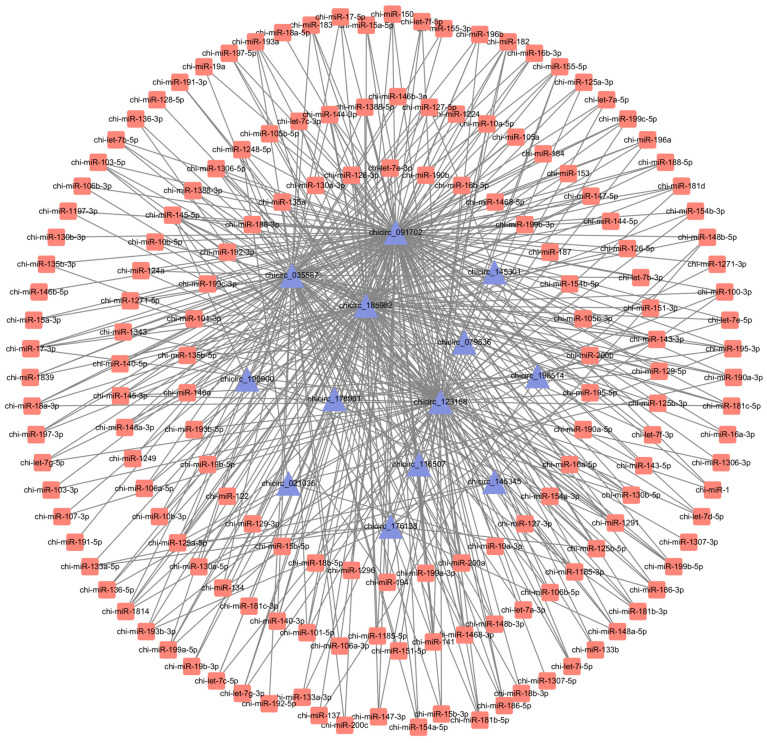
Regulatory network diagram of differentially expressed circRNAs and miRNAs.

CircRNAs can adsorb miRNAs, thereby inhibiting their functions and serving as important molecular sponges for miRNAs. Utilizing prediction results from miRanda and TargetScan, we identified 205 differentially expressed circRNAs in the hypothalamus of Chuanzhong black goats with varying reproductive abilities, which collectively target 167 miRNAs (Supplementary Table S5). On the basis of the *p*-values, we selected the top 15 differentially expressed circRNAs to construct a regulatory network of circRNAs and miRNAs (Figure 4). The results indicate that these top 15 differentially expressed circRNAs collectively target 164 miRNAs, among which miR-135a, miR-188-3p, miR-101-3p, and miR-128-3p may interact with the differentially expressed circRNAs.

### 2.5. Prediction of circRNA Coding Potential

**Figure 5:**
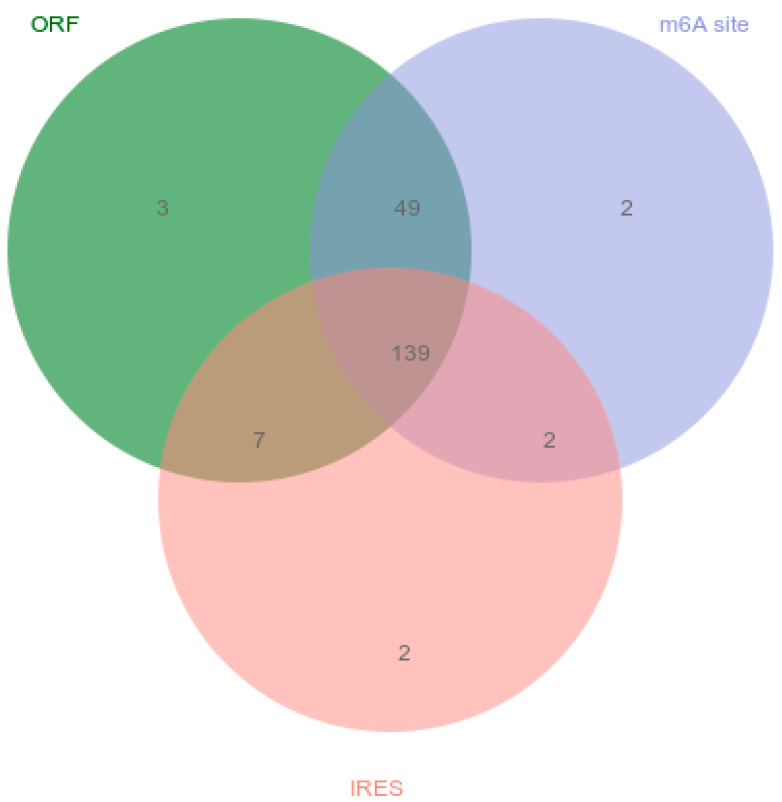
Venn diagram of circRNA coding potential predictions by ORF, SRAMP, and IRES Finder.

CircRNAs can undergo translation through primary mechanisms: the presence of IRES and the existence of m6A residues. According to predictions from the IRES finder, 150 circRNAs rely on internal ribosome entry sites, of which 26 circRNAs have a score exceeding 0.8. Additionally, predictions from the sequence-based RNA adenosine methylation site predictor (SRAMP) indicate that 192 circRNAs initiate translation dependent on m6A residues, with 78 circRNAs achieving a score greater than 0.7, which meets the criteria for very high confidence. In this study, the open reading frame (ORF) finder predicted 198 circRNAs containing ORFs among differentially expressed circRNAs. Intersecting these two translation mechanisms shows that 141 circRNAs may potentially utilize both mechanisms simultaneously (Figure 5).

### 2.6. Real-Time Fluorescence Quantitative PCR Validation

**Figure 6:**
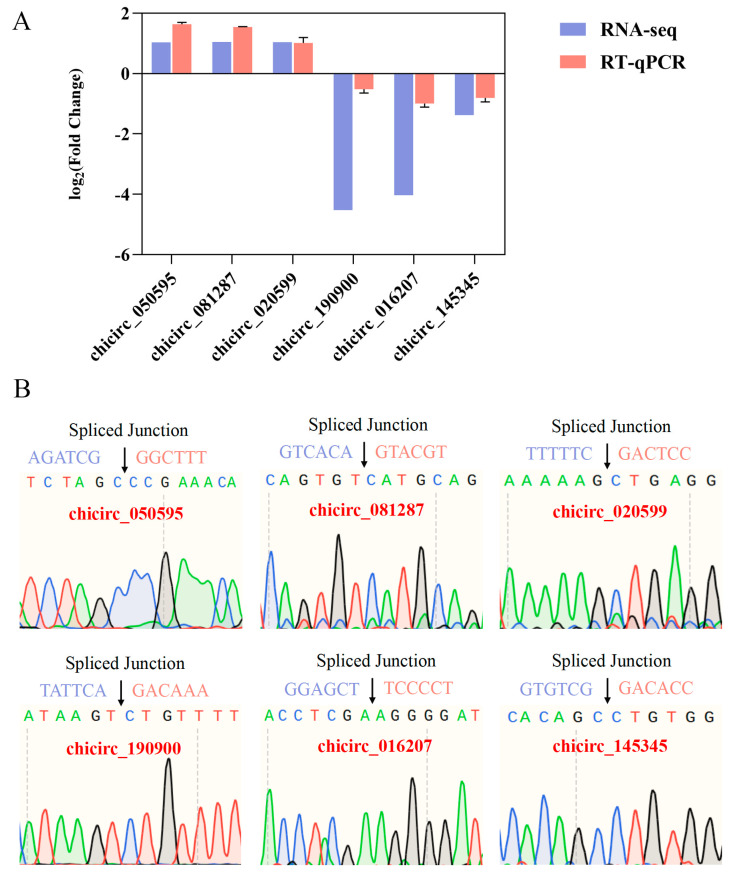
Validation of differentially expressed circRNA. (**A**) RT-qPCR validation results of differentially expressed circRNAs and (**B**) sequencing results of circularization splice sites of differentially expressed circRNAs.

RT-qPCR validation analysis was conducted on six differentially expressed circRNAs to validate the RNA-seq results (Figure 6A). The results indicate differences in expression levels between the two methods. Sanger sequencing confirmed the nucleotide sequences of the circRNA circularization splice sites. The findings demonstrated that the nucleotide sequences of these splice sites were consistent between Sanger sequencing and transcriptome sequencing (Figure 6B). The trends were aligned, suggesting the reliability of the sequencing results.

## 3. Discussion

Through high-throughput sequencing of the hypothalamus RNA in Chuanzhong black goats with low- and high-reproductive capacities, 43,295 circRNAs were identified, including 205 differentially expressed circRNAs. GO and KEGG enrichment analyses of the host genes associated with these differentially expressed circRNAs revealed significant enrichment in processes related to gonadal development, hormone secretion, steroid hormone-mediated signaling pathways, GnRH signaling pathway, oxytocin signaling pathway, and progesterone-mediated oocyte maturation. CircRNAs are a distinct class of non-coding RNA characterized by their covalently closed loop structure, which is formed through back-splicing of precursor mRNA [22]. CircRNAs exhibit high stability and are evolutionarily conserved across species because of their circular nature [23,24]. They demonstrate tissue-specific expression patterns and perform unique cellular functions [25], suggesting their potential as biomarkers for assessing the reproductive capacity of goats.

In this study, GO enrichment analysis of the host genes of 205 differentially expressed circRNAs revealed enrichment of PPP3CA, UBE3A, FOXP1, PCSK5, BRAF, and RAP1B in GO terms associated with reproductive pathways. The protein phosphatase 3 catalytic subunit alpha (PPP3CA) has been closely linked to precocious puberty [26]. A whole-genome association analysis in Dazu black goats demonstrated that PPP3CA influences estrogen signaling and oocyte meiosis [27]. Furthermore, a 20 bp insertion-deletion polymorphism in the PPP3CA gene was significantly associated with litter size in Shaanbei cashmere goats [28,29]. The circRNA-mRNA regulatory network indicates that chicirc_063269 originates from PPP3CA. Prader-Willi syndrome (PWS) is characterized by growth hormone deficiency and hypogonadism, possibly due to abnormalities in the hypothalamic-pituitary-gonadal (HPG) axis [30]. Loss of UBE3A may lead to PWS [31], as UBE3A has been shown to interact with the ubiquitin-conjugating enzyme UBCH7 to enhance progesterone receptor transactivation and promote estrogen receptor degradation [32,33]. Therefore, UBE3A may play a role in regulating litter size in goats, as indicated by the circRNA-mRNA regulatory network with chicirc_097731 as its host gene. Single-cell sequencing results revealed enrichment of FOXP2 in the pituitary gonadotroph cluster. Its loss disrupts gonadal development and affects the regulation of lhb and Fshb expression [34]. FOXP1, FOXP2, and FOXP4 can form oligomeric complexes in the brains of zebra finches [35]. FOXP1 can influence overall neural function; thus, it may affect goat reproduction by regulating the expression of lhb and Fshb, as suggested by the circRNA-mRNA regulatory network with chicirc_017440 as its host gene. When preovulatory follicles cultured in vitro were treated with LH, an increase in the mRNA and protein levels of PCSK5 was observed. PCSK5A, a subtype of PCSK5, is involved in gonadotropin regulation in the ovary and contributes significantly to ovulation by processing Pro-TGFβ and matrix metalloproteinases [36]. PCSK5 has been shown to facilitate follicular development in rats [37]. Therefore, PCSK5 may play a role in goat reproduction, as indicated by the circRNA-mRNA regulatory network with chicirc_049641 as its host gene. BRAF mRNA transcripts are localized in the central nervous system, where BRAF plays a crucial role in the development of the hypothalamic-pituitary axis in both mice and humans [38]. CREB1 can bind to BRAF to increase its expression and regulate cell proliferation [39]. BRAF is also enriched in corpus luteum-mediated oocyte maturation. Hence, BRAF may regulate goat reproduction, as indicated by the circRNA-mRNA regulatory network with chicirc_008429 as its host gene. Immunohistochemistry of cells shows that GnRH can also increase the protein levels of RAP1B [40]. RAP1B, a novel gene rapidly induced by GnRH, is a candidate gene involved in regulating gonadotropin secretion in rats [41]. According to the circRNA-mRNA regulatory network, the host gene of chicirc_145057 is RAP1B.

KEGG enrichment analysis of the host genes associated with the 205 differentially expressed circRNAs found that KIF3A, PLD1, PPP3CA, and RYR2 were significantly enriched in the GnRH signaling pathway, oxytocin signaling pathway, and progesterone-mediated oocyte maturation signaling pathway. Cilia on GnRH neurons may play a regulatory role in kisspeptin signaling [42] and can be eliminated through the conditional disruption of KIF3A [43]. Therefore, KIF3A may influence kisspeptin signaling by affecting cilia, as suggested by the circRNA-mRNA regulatory network, with chicirc_030156 and chicirc_176754 serving as host genes for KIF3A. PLD1 is known to regulate the secretion of LH and FSH in the GnRH signaling pathway [44]. Additionally, PLD1 can promote spindle assembly and migration by regulating autophagy in mouse oocytes during meiosis [45], indicating its involvement in GnRH secretion, which in turn affects LH and FSH secretion. The circRNA-mRNA regulatory network identifies chicirc_193363 as the host gene for PLD1. RYR2 plays a critical role in regulating insulin secretion and maintaining glucose homeostasis [46]. High glucose concentrations can cause irreversible damage to GnRH neurons in vitro, potentially leading to dysfunction in GnRH secretion [47]. Consequently, RYR2 may regulate GnRH secretion by modulating hypothalamic glucose concentrations [48]. This finding is supported by the circRNA-mRNA regulatory network, which identifies chicirc_030156 and chicirc_109497 as host genes for RYR2. We speculate that circRNAs play significant biological roles in regulating goat reproduction.

Through their interaction with miRNA response elements, circRNAs can indirectly enhance the transcription of their target mRNAs, thereby functioning as molecular sponges for miRNAs [49]. Notably, miR-135a, miR-188-3p, miR-101-3p, and miR-128-3p have the potential to interact with circRNAs that are produced in various ways. FSH and LH regulate the expression of the NPPC gene during oocyte meiosis by altering the protein levels of TGBR2, TGBR1, and SMAD in ovarian granulosa cells [50]. Decreased levels of the NPPC gene lead to a reduction in the synthesis of the downstream protein CNP, which, in turn, inhibits the generation of cGMP in cumulus cells. This inhibition consequently alleviates the suppressive effects on cAMP in the oocyte and triggers the resumption of meiosis [51]. Therefore, circular RNAs such as chicirc_091702, chicirc_185982, and chicirc_145345 may regulate the expression of miR-135a, which subsequently affects the production of FSH and LH and ultimately influences the meiotic process. The adsorption of miR-188-3p by circular RNAs in dental pulp stem cells leads to a decrease in Beclin-1-induced autophagy and an increase in RUNX1 expression [52,53]. The RUNX1 protein is essential for ovarian function and ovulation in rats [54]. Thus, circRNAs chicirc_091702, chicirc_185982, and chicirc_116507 may regulate goat fertility by modulating RUNX1 expression through the regulation of miR-188-3p. MicroRNA-101-3p controls the production of ZEB1, which stimulates the process of epithelial-mesenchymal transition [55]. By modulating GnRH promoter activity in the hypothalamus, ZEB1 can influence the estrous cycle in adult mice [56]. Hence, chicirc_091702 may impact the reproductive capacity of Chuanzhong black goats by regulating ZEB1 through the adsorption of miR-101-3p. MicroRNA-128-3p is crucial for the formation and functioning of the central nervous system [57]. Research has shown that it can affect the progression of glioma by regulating the signaling pathways of GREM1 and BMP [58]. Moreover, the expression of GREM1 is associated with fertility [59]; infertile individuals exhibit lower levels of GREM1 expression, while increased GREM1 expression correlates with elevated FSH production [60]. Thus, circular RNAs such as chicirc_185982, chicirc_123158, and chicirc_091702 may influence the reproductive capacity of Chuanzhong black goats by regulating miR-128-3p and the coordination between the brain, pituitary, and gonadal systems.

## 4. Materials and Methods

### 4.1. Test Animals and Sample Collection

Eleven healthy female Chuanzhong black goats, aged between 3.5 and 4.5 years, were maintained under consistent management conditions and had each given birth to three or more litters. The goats were divided into two groups: a high-prolificacy group (*n* = 6) and a low-prolificacy group (*n* = 5). The high-prolificacy group comprised goats that produced two or more offspring per litter, while the low-prolificacy group included goats that produced only one offspring per litter (Table 2). Following synchronized estrus, the 11 selected Chuanzhong black goats were slaughtered. The hypothalamus was collected and stored in cryogenic vials, rapidly frozen in liquid nitrogen, and subsequently stored long term at −80 °C.

### 4.2. RNA Extraction, cDNA Library Preparation, and Sequencing

According to the manufacturer’s instructions, total RNA was extracted from the samples using TRIzol reagent (Thermo Fisher, Shanghai, China). Ribosomal RNA was subsequently removed from the total RNA using the Ribo-Zero rRNA Removal Kit (Illumina, Inc., San Diego, CA, USA). The integrity and quantity of the RNA were assessed using the Agilent 2100 Bioanalyzer with the RNA 6000 Nano LabChip Kit (Agilent, Santa Clara, Santa Clara, CA, USA). Gel electrophoresis with 1% agarose was performed to confirm RNA integrity and the absence of genomic DNA contamination. Samples that met the quality criteria were sent to PacBio Bioinformatics (Shanghai, China) for sequencing on the Illumina HiSeq 2500 platform. For library construction, 1 μL of total RNA was utilized, and the RNA was fragmented into 200–300 bp segments. The first-strand cDNA was synthesized using random hexamers and reverse transcriptase, followed by synthesizing the second-strand cDNA. The NEBNext Ultra Directional RNA Library Prep Kit for Illumina (NEB, Ipswich, MA, USA) was employed for 150 bp paired-end sequencing in accordance with the manufacturer’s instructions.

### 4.3. Quality Assessment of Original Sequencing Data and Assembly of Transcripts

After the raw image data from the HiSeq platform were converted into FASTQ format, Cutadapt (version 1.16) was employed to control the quality of the sequence data, which involved removing adapters and low-quality reads. The filtered clean reads were then aligned to the *Capra hircus* reference genome (Genebuild by Ensembl, Genome: ARS1) using TopHat2. (version 2.1.1) Subsequently, the alignment files (in BAM format) were processed with StringTie software (version 1.3.3) to map reads to the genome and quantify transcript expression levels for each sample, as well as circRNA expression in terms of TPM (transcripts per million).

### 4.4. Screening of circRNA

To filter out lowly expressed single-exon transcripts from the transcript assembly results, we selected transcripts that contained at least two exons and had a length of ≥200 bp. Using Cuffcompare software (version 2.2.1), we identified transcripts that overlapped with annotated exonic regions in the database. After aligning the data to the reference genome, we identified circRNAs from the unmapped reads. The anchored sequences of each sample were aligned to the reference genome, and the alignment results of all samples were merged to identify circRNAs using the find_circ tool. Subsequently, we filtered highly reliable circRNAs on the basis of the following criteria: (1) retaining circRNAs with only one clear breakpoint; (2) ensuring that the overlap of the aligned positions on the genome for the anchored sequences at both ends of each read does not exceed 2 bp; (3) allowing only 2 bp mismatches; (4) ensuring that the number of unique reads is greater than two and is supported by more than half of the samples; (5) ensuring that the alignment score of at least one of the anchored sequences of each read to the genome is higher than 35; and (6) ensuring that the length of circRNA is less than 100 kb. The expression level of circRNAs was estimated in terms of TPM.

### 4.5. Differential Expression Analysis and Enrichment Analysis

Using the DESeq package in the R programming language (version 1.22.2), we conducted a differential expression analysis on the gene expression levels of the two groups. Genes with an absolute log2 fold change greater than and a significant *p*-value of less than 0.05 were identified as differentially expressed genes. The TopGO package (version 2.38.1) was utilized for GO enrichment analysis, employing the hypergeometric distribution method to identify significantly enriched GO terms (with a significance threshold set at *p* < 0.05). Additionally, KEGG pathway enrichment analysis was performed using the ClusterProfiler software (version 2.8.1), with significant enrichment defined as *p* < 0.05. On the basis of the results of the GO and KEGG enrichment analyses and their biological significance, target genes were selected for further investigation.

### 4.6. Prediction of miRNAs Targeted by circRNAs

CircRNA can adsorb miRNA, thereby inhibiting its function. The target genes of miRNAs were predicted using the miRanda (version 3.3a) and TargetScan software (version 7.2). The intersection of the predictions from both software was utilized to minimize false positive results during the prediction process. The thresholds for screening candidate miRNAs were established as TargetScan_Score ≥ 50 and miRanda_Energy < −10.

### 4.7. Prediction of circRNA Translation Potential

The ORF finder was used to identify the presence of ORFs (ORF finder; https://www.ncbi.nlm.nih.gov/orffinder/, accessed on 15 April 2024), SRAMP was used to predict the presence of m6A modification sites (http://www.cuilab.cn/sramp/, accessed on 15 April 2024), and the IRES finder was used to predict the presence of IRES (IRES finder; https://github.com/xiaofengsong/IRESfinder, accessed on 15 April 2024).

### 4.8. Real-Time Quantitative PCR Verification and DNA Sequencing Validation of RNA-Seq

According to the sequencing results, six circRNAs were selected for expression validation. GAPDH was utilized as the internal reference gene for circRNA analysis. Reverse transcription was conducted using the Takara reverse transcription kit (Takara, Kusatsu, Shiga, Japan), and gene expression levels were quantified using the 2× Ultra SYBR Green qPCR Mix (Life ABI, Astin, USA) fluorescence quantitative reagent kit. The RT-qPCR cycling parameters were pre-denaturation at 95 °C for 10 min, denaturation at 95 °C for 5 s, and annealing/extension at 60 °C for 20 s. Each experiment included three biological replicates. The relative expression levels of the target genes were analyzed using the 2^−ΔΔCt^ method. Sanger sequencing was performed on the qPCR products to verify the nucleotide sequence of the circRNA circularization splice site. The primer sequences are listed in Table S6.

## 5. Conclusions

Through high-throughput sequencing technology, we investigated the circRNA profiles of the hypothalamus in Chuanzhong black goats with varying reproductive capabilities. Our differential expression analysis revealed 205 significantly differentially expressed circRNAs. Subsequent GO and KEGG enrichment analyses of the host genes associated with these differentially expressed circRNAs, along with the construction of circRNA-miRNA regulatory networks, led to the identification of several key molecules: chicirc_063269, chicirc_097731, chicirc_017440, chicirc_049641, chicirc_008429, chicirc_145057, chicirc_030156, chicirc_109497, chicirc_030156, chicirc_176754, chicirc_193363, miR-135a, miR-188-3p, miR-101-3p, and miR-128-3p. These molecules may influence GnRH secretion and the HPG axis, thereby influencing the reproductive capabilities of Chuanzhong black goats. These findings provide a valuable reference for studying the molecular mechanisms that regulate reproduction in Chuanzhong black goats and establish a theoretical foundation for enhancing their prolificacy.

## Figures and Tables

**Table 1:** Overview of RNA sequencing of the hypothalamus in Chuangzhong black goats.

Sample	Raw Reads	Clean Reads	Clean Reads Rate /%	Multiple Mapped Rate /%	Uniquely Mapped Rate /%	Q30 /%
CZ_L1	105,651,828	104,878,170	99.26	2.38	97.62	93.33
CZ_L2	106,461,850	105,641,916	99.22	2.32	97.68	93.01
CZ_L3	103,419,368	102,762,390	99.36	1.94	98.06	93.48
CZ_L4	103,316,372	102,518,058	99.22	2.13	97.87	93.09
CZ_L5	101,666,550	100,924,246	99.26	1.45	98.55	92.51
CZ_H1	106,945,128	106,155,188	99.26	2.04	97.96	92.73
CZ_H2	103,019,774	102,205,262	99.20	1.79	98.21	92.50
CZ_H3	106,627,266	105,676,988	99.10	1.78	98.22	92.04
CZ_H4	102,279,224	101,431,548	99.17	1.63	98.37	92.29
CZ_H5	102,088,070	101,272,006	99.20	1.65	98.35	92.37
CZ_H6	101,379,018	100,570,708	99.20	2.13	97.87	92.43

**Table 2:** Chuanzhong black goat lambing record.

Serial Number	Date of First Delivery	Number of Lambs in the First Litter	Date of Second Delivery	Number of Lambs in the Second Litter	Date of Third Delivery	Number of Lambs in the Third Litter	Reproductive Condition
CZ_L1	10 November 2016	1	17 July 2017	1	12 April 2018	1	low fecundity
CZ_L2	14 November 2016	1	16 July 2017	1	20 April 2018	1	low fecundity
CZ_L3	28 November 2016	1	9 August 2017	1	11 May 2018	1	low fecundity
CZ_L4	1 October2016	1	16 September 2017	1	27 April 2018	1	low fecundity
CZ_L5	19 December2016	1	7 September 2017	1	2 June 2018	1	low fecundity
CZ_H1	3 January 2017	2	31 August 2017	3	22 March 2018	2	high fecundity
CZ_H2	5 September 2016	2	24 February 2017	2	21 December 2017	3	high fecundity
CZ_H3	16 July 2016	2	14 February 2017	2	15 January 2018	2	high fecundity
CZ_H4	11 November 2016	3	28 July 2017	2	22 February 2018	2	high fecundity
CZ_H5	20 November 2016	2	30 June 2017	2	10 February 2018	3	high fecundity
CZ_H6	18 November 2016	2	24 July 2017	3	19 February 2018	2	high fecundity

## Data Availability

The raw sequence data reported in this paper have been deposited in the Genome Sequence Archive (Genomics, Proteomics & Bioinformatics 2021) in National Genomics Data Center (Nucleic Acids Res 2022), China National Center for Bioinformation/Beijing Institute of Genomics, Chinese Academy of Sciences (GSA: CRA016713) that are publicly accessible at https://ngdc.cncb.ac.cn/gsa, accessed on 27 May 2024.

## Supplementary Materials

The supporting information can be downloaded at: https://www.mdpi.com/article/10.3390/ijms251910479/s1.
